# Effect of Sand-Frying-Triggered Puffing on the Multi-Scale Structure and Physicochemical Properties of Cassava Starch in Dry Gel

**DOI:** 10.3390/biom11121872

**Published:** 2021-12-14

**Authors:** Yonglin He, Fayin Ye, Sheng Li, Damao Wang, Jia Chen, Guohua Zhao

**Affiliations:** 1 College of Food Science, Southwest University, Chongqing 400715, China; ylh1993@email.swu.edu.cn (Y.H.); fye@swu.edu.cn (F.Y.); lisheng1990@email.swu.edu.cn (S.L.); wangdamao@swu.edu.cn (D.W.); swaumyh@swu.edu.cn (J.C.); 2 Chongqing Engineering Research Center for Sweet Potato, Chongqing 400715, China

**Keywords:** cassava starch gel, puffing process, multi-scale structure, physicochemical properties

## Abstract

This study revealed the underlying mechanisms involved in the puffing process of dried cassava starch gel by exploring the development of the puffed structure of gel upon sand-frying, chiefly focused on the changes in the multi-scale structure and the physicochemical properties of starch. The results suggested that the sand-frying-induced puffing proceeded very fast, completed in about twenty seconds, which could be described as a two-phase pattern including the warming up (0~6 s) and puffing (7~18 s) stages. In the first stage, no significant changes occurred to the structure or appearance of the starch gel. In the second stage, the cells in the gel network structure were expanded until burst, which brought about a decrease in moisture content, bulk density, and hardness, as well as the increase in porosity and crispness when the surface temperature of gel reached glass transition temperature of 125.28 °C. Upon sand-frying puffing, the crystalline melting and molecular degradation of starch happened simultaneously, of which the latter mainly occurred in the first stage. Along with the increase of puffing time, the thermal stability, peak viscosity, and final viscosity of starch gradually decreased, while the water solubility index increased. Knowing the underlying mechanisms of this process might help manufacturers produce a better quality of starch-based puffed products.

## 1. Introduction

As is well known, starch is a versatile ingredient widely used in various foods as a thickener, stabilizer, or a gelling agent. Starch is also the most important nutrient, which provides the major energy for the human body [1]. More importantly, many snacks are fabricated from starch or starchy foodstuffs, such as shrimp crackers, popcorn, and crunchy rice candy [2,3,4]. Due to their pleasant texture, nice flavor, and high convenience, the consumption of starch-based snacks has climbed in recent years [5]. To ensure the high quality of starch-based snacks, puffing methods such as extrusion puffing, hot air puffing, microwave puffing, oil frying puffing, gun puffing, and sand- or salt-frying puffing, were adopted as a critical procedure in their production [6]. By these means, a serial of high quality puffed products was produced from cereal grains (wheat, oat, rye, barley, rice, black rice, glutinous rice, maize, sorghum, millet, and buckwheat) [7,8,9], amaranth seeds [10], quinoa seeds [11], chickpea [12], banana slices [13], mango chips [14], and fish crackers [15].

Besides the puffing regime, starch possessing the appropriate structure is usually applied to develop high-quality starch-based puffed snacks. For instance, Joshi et al. selected the rice varieties (Gurjari, Jaya, GR-5, and GR-6) to produce superior puffed products, which was credited by their having the proper ratio of amylose to amylopectin [16]. Dias et al. utilized sodium hypochlorite to modify the structure of cassava starch, producing biscuits with high expansion characteristics [17]. It has been reported by Junior et al. that sour cassava starch generated by fermentative treatment exhibited excellent puffing ability in creating its puffed products [18]. Some external additives, such as fruit pomace (apple, cranberry, blueberry, and grape), microalgae powder, and high methoxyl pectin, could be adapted to enhance the puffing performance of starch-based snacks [19,20,21]. Optimizing process parameters, such as moisture content, heating temperature, and heating time, also contributed to enhancing the quality of puffed products [11,22].

A clear understanding of the underlying mechanisms involved in the puffing process is needed for obtaining a better quality of puffed products [6]. Currently, some scholars are trying to use physics-based modeling and experiments to clarify the underlying mechanisms of the puffing process of rice, but they have not considered the changes in multi-scale structure and physicochemical properties of rice starch coincident with the increase of puffing time [6]. In addition, some researchers only study the alterations of multi-scale structure and physicochemical properties of rice starch before and after puffing, but are not involved in variations at varying moments of puffing related to the whole puffing process. In addition, the underlying mechanisms involved in the puffing process of rice were not revealed [23,24,25,26]. Therefore, this work aimed to systematically unravel the underlying mechanisms of the puffing process of dried cassava starch gel by exploring the development of the puffed structure of gel upon sand-frying, chiefly concentrated on the changes in the multi-scale structure and the physicochemical properties of starch. Although this work only involved the puffing process of cassava starch gel, its puffing process was quite similar to other starch gels. The quality of starch-based puffed snacks is mainly dependent on the puffing performance of a starch gel. Accordingly, this work could help manufacturers produce a better quality of starch-based puffed products.

## 2. Materials and Methods

### 2.1. Materials

Cassava starch was purchased from Guangxi Honghao Starch Development Co., Ltd. (Nangning, Guangxi, China). Its proximate composition was measured by AACC methods, namely starch (76-11), amylose (61-03), crude protein (46-13), crude lipid (30-25), moisture (44-15A) and ash (08-17) [27]. The results were indicated in Table S1. Fine sand was bought from Qingyuan Jinfeng River Sand Co., Ltd. (Qingyuan, Guangdong, China). Isoamylase (1000 U/μL), sodium acetate (purity ≥ 99.0%), sodium azide (purity ≥ 99.5%), lithium bromide (purity ≥ 99.0%), and dimethyl sulfoxide (purity ≥ 99.9%) were obtained from Sigma Chemical Co., Ltd. (St. Louis, MO, USA).

### 2.2. Preparation of Dried Cassava Starch Gel and Its Puffing Operation

Cassava starch (40 g) was suspended in water (50 mL), and the resultant slurry was poured into a stainless tray (21.0 × 16.5 × 2.50 cm). The slurry was spread uniformly on the bottom of the tray by gently shaking it, and then the tray was horizontally floated over a boiling water bath for 15 s. Subsequently, the tray was immersed in boiling water for approximately 30 s. After that, the tray was immediately removed and then immersed in a tap water bath for approximately 15 s to cool down. As a result, a starch gel formed on the bottom of the tray [27]. The gel sheet was carefully removed from the tray and stored at 4 °C for 24 h. After storage, the gel sheet was shaped into discs having a diameter of 4.8 cm. The discs were dried in an oven at 50 °C until a moisture content of 12% was attained, which resulted in dried starch discs weighing approximately 2.2 g each (with a diameter of 4.0 cm and a thickness of 2.5 mm).

To puff the dried starch discs, fine sand (300 g) was loaded in an iron pan and heated to 200 ± 10 °C with the aid of an induction cooker (GW-30D6, Gudves, Zhongshan, Guangdong, China). The temperature of the sand bed was measured using an AS842A infrared thermometer (Smart Sensor, Guangdong, China). Then starch discs were fed into a hot sand bed and quickly blended with a stainless steel spatula to achieve uniform frying. A video of puffed starch gel preparation was provided as Video S1. However, in this study, the starch discs were sampled at frying intervals (0, 3, 6, 9, 12, 15, and 18 s) and hermetically packaged in polyethylene bags as they were cooled to room temperature. All samples were stored in a desiccator awaiting further measurements.

### 2.3. Evaluation of Puffing Performance of Dried Cassava Starch Gel

The puffing performance of the dried starch discs was evaluated in terms of puffing ratio, moisture content, bulk density (*p_b_*), true density (*p*), porosity (ε), hardness, crispness, and color. The volume of the starch disc was determined by a fine sand replacement method, and the puffing ratio was defined as the ratio of disc volume before and after puffing [22]. The moisture content was measured by oven drying at 105 °C. The bulk density (*p_b_*, g/mL) was obtained by dividing the disc’s mass by its volume. The true density (*p*) was determined by a toluene displacement method as described previously by Joshi et al. [16]. The porosity (ε) was calculated as (*p*−*p_b_*)/*p* × 100%. The hardness and crispness were determined by a puncture test method with the aid of a TA.XT plus texture analyzer (Stable Micro Systems, Godalming, UK) equipped with a spherical probe (P/0.5s) [28]. A trigger force of 5 g, as well as pretest, test, and return rates of 1.0 mm/s, 1.0 mm/s, and 10 mm/s were applied. As a result, the first and the maximum peak forces breaking the disc represented the crispness and hardness, respectively [14,29]. The lower value of the first peak force indicates higher crispness. The color of the starch disc was measured using a colorimeter (UltraScan PRO, HunterLab, Reston, VA, USA). Prior to the test, the instrument was calibrated with a whiteboard and blackboard.

### 2.4. Characterization of Microstructure of Dried Starch Gel

The cross-sectional images of starch discs were recorded by scanning electron microscopy (SEM) (Phenom Pro, Phenom World, Eindhoven, The Netherlands) at an accelerating voltage of 10 kV. The pore size distribution was determined with a mercury porosimeter (AutoPoreV, Micromeritics Instrument Corporation, Norcross, GA, USA) with pressure varied from 0.1 to 61,000 psia. Before measurement, the sample (1 cm^2^, approximately 50 mg) was first put into a dilatometer and then evacuated.

### 2.5. Determination of Physicochemical Properties of Starch in Discs

To determine the physicochemical properties of starch, the discs were firstly ground and passed through a 200-mesh sieve. The water solubility index (WSI) was measured according to the method described by Kaisangsri et al. [30]. The pasting properties were determined by a rapid viscosity analyzer (RVA-TecMaster, Perten Instruments, Stockholm, Sweden) and the parameters of pasting temperature (PT_emp_), peak time (PT), peak viscosity (PV), trough viscosity (TV), final viscosity (FV), breakdown (BD), and setback (SB) were recorded [31].

The thermal stability was analyzed by a thermogravimetric analyzer (TA Instruments, New Castle, DE, USA) under nitrogen by heating the samples from 30 °C to 600 °C at a rate of 10 °C/min. The glass transition temperature (*T*_g_) of the starch disc was evaluated under tensile mode using a Q800 Dynamic Mechanical Analyser (TA Instruments LLC, New Castle, DE, USA). The samples were heated from 30 °C to 200 °C with a heating rate of 5 °C/min. The oscillating frequency was set at 1 Hz. In this study, the temperature corresponding to the peak value of tan δ curve was taken as *T*_g_.

### 2.6. Characterization of the Ordered Structure of Starch in Discs

The ordered starch structure in the discs was analyzed by an X’Pert3 Powder X-ray diffractometer (PANalytical, Amsterdam, The Netherlands) and a Spectrum100 FT-IR spectrometer (Perkin-Elmer, Waltham, MA, USA). The diffractometer was operated at 40 kV and 40 mA across the diffraction angle range of 4°(2*θ*)–40°(2*θ*) at a scanning rate of 2°/min. The MDI-Jade 6.0 software (Material Date, Sacramento, CA, USA) was utilized to calculate the relative crystallinity. For FT-IR, the obtained spectrum was processed by deconvolution using a PeakFit software (version 4.12, SeaSolve Software Inc., Boston, MA, USA) [32].

### 2.7. Determination of Molecular Structure of Starch in Discs

The molecular weight of the starch was determined according to a previous method with some modifications [33]. The starch powder (5.0 mg) was dissolved in DMSO containing 5 mM LiBr (2 mL) by heating in a boiling water bath. After being cooled to room temperature, the starch solution was centrifuged at 12,000× *g* for 10 min, and the resulting supernatant was filtered through a 0.22 μm PTFE membrane. An aliquot of the filtrate (100 μL) was applied onto a high-pressure size exclusion chromatography system (Waters, Milford, MA, USA), coupled with a multi-angle laser light scattering detector (DAWN HELEOS Ⅱ, Wyatt Technology, Santa Barbara, CA, USA) and a refractive index detector (Optilab T-rEX, Wyatt Technology, Santa Barbara, CA, USA). A set of chromatographic columns connected in series, i.e., Ohpak SB-805 HQ, Ohpak SB-804 HQ, and Ohpak SB-803 HQ (Shodex, Tokyo, Japan), were used to separate. DMSO containing 5 mM LiBr was used as the eluent, and the flow rate was controlled at 0.3 mL/min. Astra 5.3.4.20 software (Wyatt technology, Santa Barbara, CA, USA) was used to analyze the collected data from light scattering. The weight-average molecular weight (*M_w_*) and number-average molecular weight (*M_n_*) were calculated using the second-order berry method.

The chain length of amylopectin molecules was analyzed with high-performance anion-exchange chromatography coupled to a pulsed amperometric detector. Briefly, fractionated amylopectin (5.0 mg) was suspended in 5 mL of double-distilled water and heated in a boiling water bath for 60 min with an intermittent vortex. The sodium acetate buffer (50 μL, 0.6 M, pH 4.4), NaN3 (10 μL, 2% *w*/*v*) and 5 μL of isoamylase (1000 U/μL) were added to 2.5 mL of a gelatinized sample and stirred for 24 h at 37 °C. Then, the mixture was heated for 5 min to deactivate the enzymes. After placing 600 μL of the mixture in a centrifuge tube, it was dried by blowing nitrogen at room temperature. Finally, the dried sample was dissolved in 600 μL of mobile phase, centrifuged at 12,000× *g* for 5 min, and 20 μL of supernatant was analyzed by HPAEC-PAD. The ICS5000 system (Dionex Corporation, Sunnyvale, CA, USA) equipped with a 4 × 50 mm CarboPac PA1 guard column and a 4 × 250 mm Dionex™ CarboPac™ PA10 analytical column was used. The sample was eluted with a mixture of 0.2 mol/L NaOH and 0.2 mol/L NaAC. The flow rate was set at 0.5 mL/min. PeakNet (Dionex, Sunnyvale, CA, USA) was used to analyze the experimental data.

### 2.8. Statistical Analysis

The tests were performed at least three times unless otherwise specified. The data were evaluated using analysis of variance (ANOVA) by SPSS statistical software (ver.17.0, SPSS Inc, Chicago, IL, USA). A comparison of means was carried out using Duncan’s test. A *p* < 0.05 was considered statistically significant.

## 3. Results and Discussion

### 3.1. The Changes of Physical Properties of Dried Starch Gel upon Puffing

The alterations of appearance, puffing ratio, porosity and moisture content, color, and textural properties of starch gel in the sand-frying puffing process were investigated, and associated results were displayed in Figure S1 and Table 1. It could be observed that during the heating time from 0 to 6 s (heating stage), there was essentially no variation in the physical characteristics of the gel (*p* > 0.05). The puffing ratio, porosity, whiteness (L*), total color difference (ΔE*), and crispness of the gel were progressively increased from beginning at 7 s (puffing stage), whereas the moisture content, redness (a*), yellowness (b*), and hardness were decreased step by step. Finally, the physical properties of the gel were not altered fundamentally. At the heating stage, the surface temperature of the gel did not reach its glass transition temperature (*T*_g_) (Figure S2), so the gel could not be puffed. It has been proven that a phase transition from a glassy state (rigid) to a rubbery state (soft) was necessary for material expansion [34]. In addition, there was not enough pressure to expand the gel since the water remained largely unevaporated. Therefore, these physical properties did not vary. However, in the puffing stage (7~18 s), the gel experienced the phase transition from glassy state to rubbery state and a strong internal pressure was generated in the gel due to the large amount of water evaporation. This caused the changes of the physical characteristics, and the evolution of textural properties could be attributed to the porous structure of the gel upon sand-frying as this structure required only a low force to break it [35]. In addition, this porous structure enhanced the light flux, which increased the whiteness of the gel [34].

### 3.2. The Microstructural Evolutions of Dried Starch Gel upon Puffing

**Figure 1:**
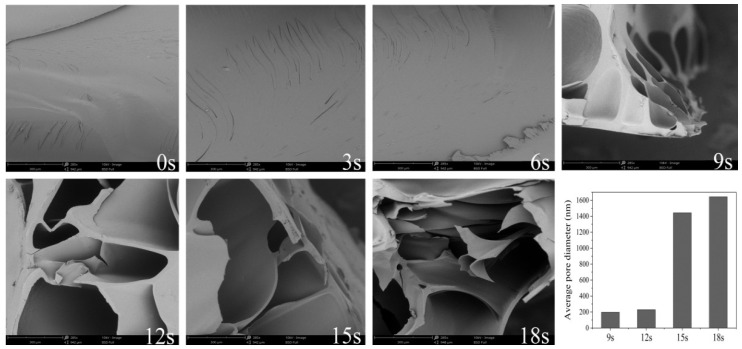
The cross-sectional microstructure and mean pore diameter of dried cassava starch gel at various puffing times by sand-frying.

The cross-sectional microstructure of gels at different puffing moments are pre-sented in Figure 1. The results indicate that during the heating stage, the microstructure of gels had no pore. Nevertheless, in the puffing stage, its microstructure showed a lot of small uniform pores and pore size was gradually enhanced along with the increase of puffing time. This was due to the gel turning to the rubbery state from the glass state. Subsequently, the excessive pressure formed via the steam caused the rupture of the pore walls in gel, thereby generating larger pores. Some researchers reported this similar phenomenon in the sand-frying puffing process of rice. They found that as puffing proceeded, pore walls became thinner. When the stress caused by gas pressure surpassed the failure, the stress on the rice resulted in pore rupture, thus generating bigger pores [6].

It can be seen from Figure S3 that large and small pores coexisted in gels at the puff moments. In addition, the mean pore diameter of the above mentioned puffed gels were 197.70, 229.71, 1443.54, and 1643.48 nm, respectively, suggesting that more and more big pores were formed in the gel under the sand-frying puffing process, which would increase the porosity of the gel.

### 3.3. The Puffing-Induced Changes in the Physicochemical Properties of Starch

The water solubility index represents the number of small molecules dissolved in water [20]. As illustrated in Table 1, the WSI of cassava starch sharply increased (*p* < 0.05) within the heating stage. However, in the puffing stage, the WSI of cassava starch increased slowly. This might be caused by the large degree of degradation of cassava starch in the heating stage. Nevertheless, cassava starch was only degraded slightly during the puffing stage [26]. These results revealed that hierarchical degradation of cassava starch occurred in the sand-frying puffing process. Huang et al. also found that the WSI of millet, barley, wheat, rice, black rice, and glutinous rice increased significantly after explosion puffing, mainly due to the degradation of starch [8].

**Figure 2:**
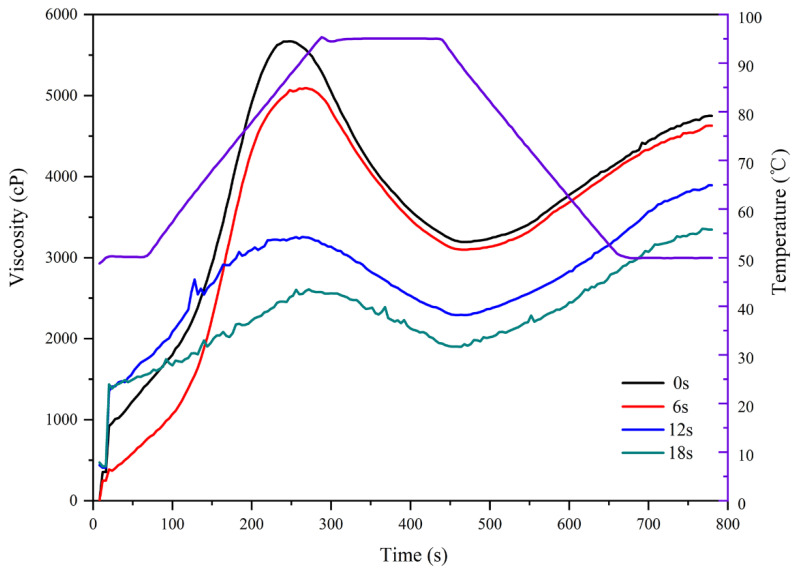
The pasting curves of cassava starch at varying moments of puffing by sand-frying.

The pasting properties can shed light on the alterations in viscosity of starch suspension when heated, and reflect the changes of starch structure. The pasting curves (Figure 2) and its corresponding parameters of cassava starch at various puffing times are exhibited in Table 2. The results showed that the pasting temperatures were of no significant difference (*p* > 0.05) in all samples. The peak time ranged from 4.16 to 4.53 min. The final viscosity, peak viscosity, trough viscosity, setback, and breakdown ranged from 3256 to 4730 Cp, from 2639 to 5655 Cp, from 1910 to 3226 Cp, from 1346 to 1818 Cp, and from 729 to 2429 Cp, respectively. It is worth noting that both peak viscosity and final viscosity were markedly reduced (*p* < 0.05) in the sand-frying puffing process, which mainly occurred in the puffing stage of the starch gel. It has been reported that both peak viscosity and final viscosity of grain (wheat, rye, barley, and buckwheat) starch in the gun puffing process were reduced [36]. Lai and Cheng also found this similar phenomenon in the hot air or gun puffing process of rice starch [37]. Hidalgo et al. also confirmed that the peak viscosity and final viscosity of wheat starch were drastically decreased after gun puffing [38]. The decrease of peak viscosity might be associated with the damage of residual starch granules because granular swelling was a major contributor to the peak viscosity. Furthermore, because starch has more branched amylopectin, it can absorb more water and exhibit a higher peak viscosity [39]. Therefore, the decrease of peak viscosity of cassava starch might also be due to the destruction of its amylopectin structure. However, the leached amylose and amylopectin from starch granules contributed to the final viscosity of starch. As a consequence, the reduced final viscosity might be closely related to the destruction of molecular structure.

**Figure 3:**
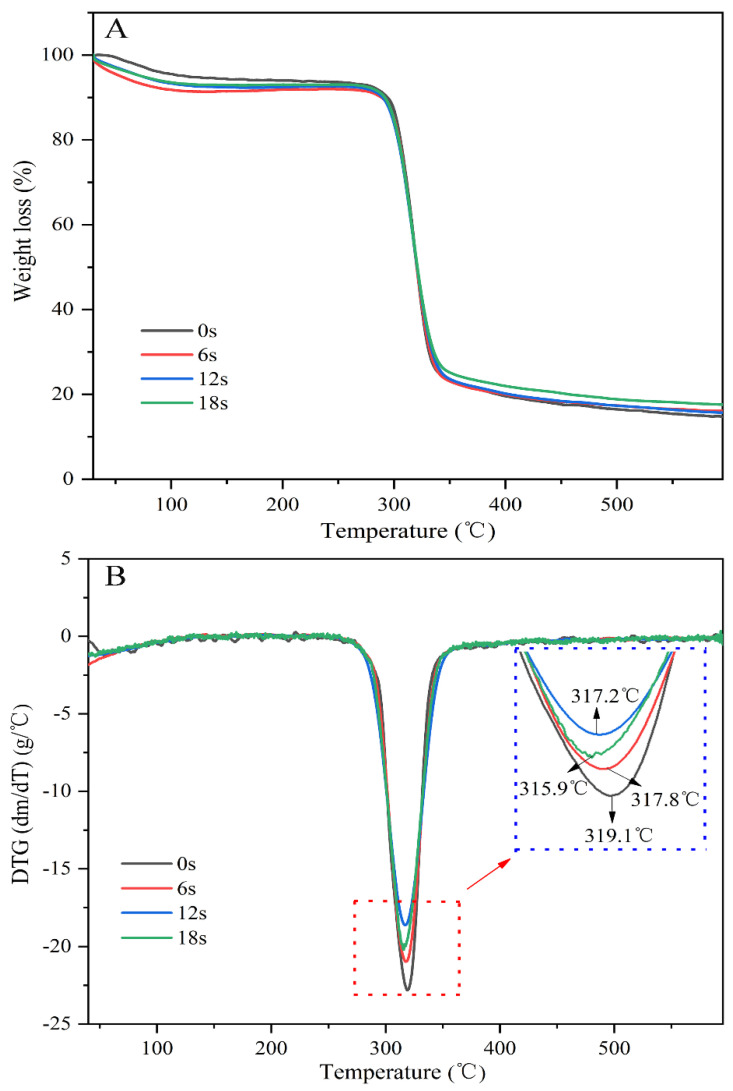
(**A**) Thermogravimetric (TG), and (**B**) corresponding derivative TG (DTG) curves of cassava starch under various puffing times by sand-frying.

The thermal stability of cassava starch under different puffing moments was evaluated, and relevant results, including thermogravimetric (TG) and corresponding derivative TG (DTG) curves, are presented in Figure 3. In this study, there were two major weight loss points. The first weight loss (<120 °C) was caused by losing water molecules. The second weight loss (>260 °C) was attributed to the pyrolysis of cassava starch [40]. It was difficult to estimate the thermal stability of cassava starch under various puffing times from the TG curves. However, DTG curves showed that the temperatures linked to the biggest weight loss rate gradually decreased with the increase of puffing time, revealing that the thermal stability of cassava starch was reduced in the sand-frying puffing process (heating stage and puffing stage). In 2020, Zapana et al. studied the effects of three puffing methods, including gun puffing, extrusion puffing, and microwave puffing on the thermal stability of quinoa flour. They found that compared with raw quinoa flour, quinoa flour puffed by the three puffing methods mentioned above possessed lower thermal stability [11]. This could be caused by the alterations in the multi-scale structure of starch.

### 3.4. The Puffing-Induced Changes in the Multi-Scale Structure of Starch

**Figure 4:**
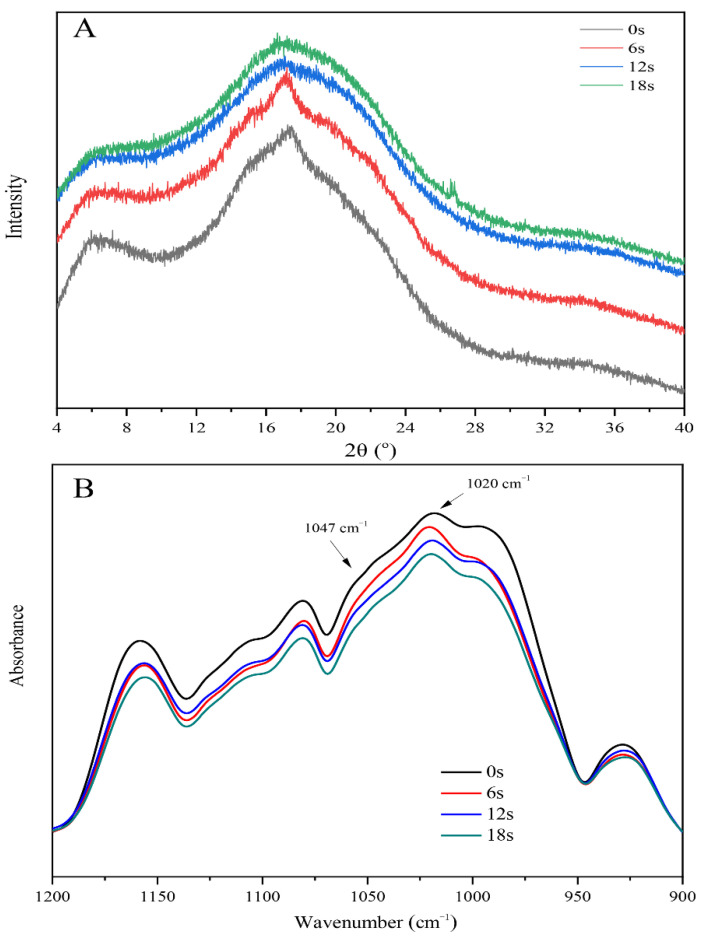
(**A**) X-ray diffraction patterns, and (**B**) the deconvoluted infrared spectra of cassava starch under different puffing times by sand-frying.

The XRD spectra of cassava starch under various puffing times were presented in Figure 4A. The results indicated that cassava starch has two diffraction peaks at 2*θ* = 5.6° and 2*θ* = 17° at the puffing time of 0 s. This was a typical B-type crystallinity pattern. With an increase of puffing time, the diffraction peaks at 2*θ* = 17° would disappear, and the intensity of diffraction peaks at 2*θ* = 5.6° was reduced. These results suggested that puffing might be closely related to the diffraction peak of 2*θ* = 17°. In addition, the long-range crystalline structure of cassava starch was transformed into an amorphous structure in the sand-frying puffing process [41]. This point was further confirmed by the data of relative crystallinity, as shown in Table 2. At present, it has been reported that rice starch experienced the transformation from crystalline structure to amorphous structure in the sand-frying puffing process [26,42]. In 2018, the study of Kristiawan et al. also suggested that pea starch lost its crystalline structure in the extrusion puffing process [24]. Similarly, it has been revealed that extrusion puffing could lead to the cleavage of intermolecular and intramolecular hydrogen bonds, thereby causing the dissociation of the helical structure. This would destroy the crystalline structure of fava bean, black bean, and pinto bean starches [43]. In this study, it is worth noting that in the heating stage, cassava starch possessed a certain degree of relative crystallinity. However, in the puffing stage, cassava starch had very low relative crystallinity. This was because a stronger starch gel network formed when it possessed a certain degree of crystallinity, conducive to holding more vapor in the heating stage. Whereas in the puffing stage, low relative crystallinity helped expand the starch gel network.

Figure S4 shows that cassava starch experienced no changes regarding the shape and position of the peak under the wavenumber range of 4000 to 500 in the sand-frying puffing process, revealing that puffing could not cause the formation of new functional groups [44]. The deconvoluted infrared spectra of 1200–900 cm^−1^ was obtained as presented in Figure 4B, of which 1047 cm^−1^ represented molecular order and crystallinity of starch. 1020 cm^−1^ represented the amorphous or disorder phase of starch. The ratio of 1047/1020 cm^−1^ can be used to quantify the molecular order degree of starch [45]. As shown in Table 2, the ratio of 1047/1020 cm^−1^ was gradually reduced as the puffing time increased, indicating that the molecular order degree of cassava starch decreased during the sand-frying puffing process, which primarily occurred in the heating stage. A similar result has been reported by Kuo et al. [25]. They discovered that the ratio of 1047/1020 cm^−1^ was reduced in the steam cooking or extrusion puffing process, thus influencing the molecular order degree of rice starch. These results indicate that the low molecular order degree of starch might be conducive to puffing.

**Figure 5:**
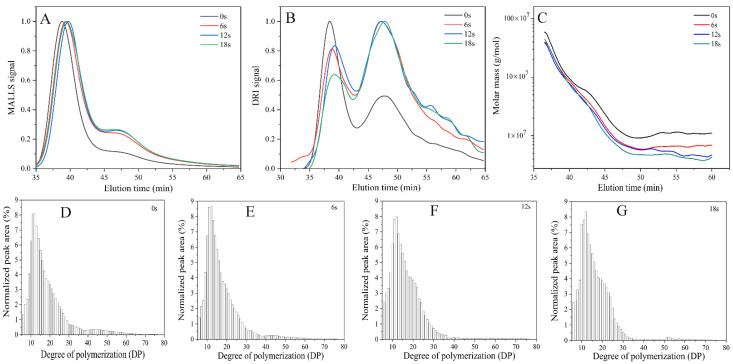
(**A**,**B**) the SEC-MALLS-DRI chromatograms of cassava starch under various puffing times by sand-frying; (**C**) the profiles of molar mass versus elution time of cassava starch at different puffing times by sand-frying. The chain length distribution of cassava amylopectin by sand-frying under puffing times of 0 s (**D**), 6 s (**E**), 12 s (**F**), and 18 s (**G**).

The SEC–MALLS–DRI chromatograms and graphs of molecular weight versus elution time of cassava starch under various puffing times were obtained, as illustrated in Figure 5. It can be observed from Figure 5A,B that as the puffing time increased, both LS and RI signals at the first peak were slightly delayed. In addition, the intensity of LS and RI signals at the second peak increased. These results suggested that the molecular weight of cassava starch was degraded in the sand-frying puffing process, which was further proved by graphs of molecular weight versus elution time (Figure 5C). The values of *M_w_* and *M_n_* are summarized in Table 3. It can be seen that both *M_w_* and *M_n_* were decreased with the increase of puffing time. Liu et al. also found that the degradation of the molecular weight of corn starch occurred during the puffing process [46]. In this work, it was interesting that an obvious degradation of the molecular weight of cassava starch had appeared in the heating stage of gel. However, in the puffing stage of the gel, the molecular weight of cassava starch only occurred during the small degradation. These results implied that the destruction of the molecular structure in the heating stage might be beneficial in the puffing stage.

The chain length distribution of cassava amylopectin at different puffing times were presented in Figure 5D–G and Table 3. Amylopectin branch chains are usually classified into four parts: chain A (DP 6–12), chain B_1_ (DP 13–24), chain B_2_ (DP 25–36), and chain B_3_ (DP ≥ 37) [47]. The results indicated that with the increase of puffing time, the relative proportion of chain B_2_, chain B_3_, and average chain length (*CL*) decreased from 11.1% to 8.2%, from 6.9% to 2.6%, and from 18.4 to 16.6, respectively. However, there was an increase in the relative proportion of chain A and chain B_1_ from 32.3% to 35.9%, and from 49.7% to 53.3%, which proved that cassava amylopectin was degraded in the sand-frying puffing process, thus reducing the molecular weight of cassava starch. Li et al. also reported the analogous findings. They discovered that the relative proportion of the A chains (21.1%) of potato amylopectin remained unchanged when it was treated with moisture-heat treatment (25.7% moisture content, 120 °C), but that the relative proportion of B_1_ chains increased from 49.1% to 49.6%, and those of the B_2_ and B_3_ chains were reduced from 17.8% to 17.6%, and from 12.0% to 11.6%, respectively [48]. Due to sand-frying puffing having a higher temperature than moisture-heat treatment, cassava amylopectin degraded more severely than potato amylopectin. It was noteworthy to this study that the degradation of cassava amylopectin occurred simultaneously with the crystalline melting and the destruction of the short-range ordered structure of cassava starch. These consequences demonstrated that the puffing of starch gel was closely related to variations of the multi-scale structure of starch.

## 4. Conclusions

In this study, the underlying mechanisms involved in the puffing process of dried cassava starch gel were revealed. The results indicated that the puffing of starch gel was a complex process, and the whole process was mainly divided into two stages, namely the heating stage and the puffing stage. In the heating stage (0~6 s), starch gel experienced no significant changes in its appearance and structure. In the puffing stage (7~18 s), the cells in the gel network structure expanded until they burst, which caused changes in the physical properties of gel when the surface temperature of the gel reached glass transition temperature. The destruction of the crystalline structure and the short-range molecular order structure of cassava starch and molecular degradation occurred simultaneously in the above two stages, with molecular degradation mainly occurring in the first stage. Meanwhile, the thermal stability, peak viscosity, and final viscosity of the starch gradually decreased, while the water solubility index increased along with the enhancement of puffing time. Although this work only figured out the underlying mechanisms for the puffing process of cassava starch gel, the physics of the process were quite similar to other starch gels. Therefore, this work might help manufacturers produce a better quality of starch-based puffed products. However, it must be noted that the present study only involved the puffing of starch gel related to the time scale, whereas the puffing of starch gel concerning moisture and temperature scales has not been conducted. This should be addressed in subsequent investigations.

## Figures and Tables

**Table 1:** The physical properties of dried cassava starch gel at varying puffing times by sand-frying.

Parameters ^a^	Puffing Time (s)						
	0	3	6	9	12	15	18
Puffing ratio	1.00 ± 0.02 ^e^	1.01 ± 0.04 ^e^	1.16 ± 0.03 ^e^	4.38 ± 0.38 ^d^	7.07 ± 0.26 ^c^	10.26 ± 0.53 ^b^	10.69 ± 0.62 ^b^
Moisture (g/100 g)	12.33 ± 1.19 ^b^	12.23 ± 1.21 ^b^	11.97 ± 1.35 ^b^	8.04 ± 0.18 ^c^	6.37 ± 1.19 ^cd^	4.08 ± 0.65 ^d^	3.25 ± 0.05 ^de^
Porosity (%)	5.77 ± 1.54 ^f^	5.89 ± 0.88 ^f^	5.96 ± 1.04 ^f^	41.11 ± 8.40 ^e^	71.29 ± 1.37 ^d^	84.55 ± 1.19 ^c^	89.04 ± 0.89 ^b^
Surface temperature (°C)	31.67 ± 1.53 ^f^	111.67 ± 3.06 ^e^	129.33 ± 4.93 ^d^	162.67 ± 4.51 ^c^	177.33 ± 2.08 ^b^	181.33 ± 2.52 ^b^	181.67 ± 4.93 ^b^
L*	37.82 ± 1.86 ^e^	37.78 ± 2.11 ^e^	38.21 ± 1.72 ^e^	53.29 ± 1.38 ^d^	61.81 ± 0.59 ^c^	67.47 ± 2.15 ^b^	66.84 ± 1.35 ^b^
a*	3.08 ± 0.18 ^b^	3.09 ± 0.40 ^b^	3.09 ± 0.31 ^b^	2.41 ± 0.14 ^c^	1.76 ± 0.08 ^d^	1.47 ± 0.09 ^e^	1.35 ± 0.04 ^e^
b*	6.39 ± 0.30 ^b^	6.27 ± 0.12 ^b^	6.26 ± 0.45 ^bc^	5.62 ± 0.29 ^cd^	5.42 ± 0.26 ^d^	4.84 ± 0.20 ^e^	4.64 ± 0.55 ^e^
*C*	7.11 ± 0.35 ^b^	7.01 ± 0.28 ^b^	6.99 ± 0.33 ^b^	6.11 ± 0.32 ^c^	6.02 ± 0.34 ^c^	5.07 ± 0.19 ^d^	4.84 ± 0.54 ^d^
*H*	64.23 ± 0.37 ^d^	63.79 ± 2.61 ^d^	63.64 ± 3.56 ^d^	66.81 ± 0.95 ^c^	71.93 ± 1.31 ^b^	73.08 ± 1.12 ^b^	73.63 ± 1.47 ^b^
ΔE*	−	1.94 ± 0.89 ^d^	2.66 ± 0.77 ^d^	15.52 ± 0.72 ^c^	24.05 ± 1.74 ^b^	29.73 ± 3.74 ^b^	29.13 ± 2.93 ^b^
Bulk density (g/mL)	2.69 ± 0.09 ^b^	2.80 ± 0.10 ^b^	2.79 ± 0.05 ^b^	0.60 ± 0.04 ^c^	0.38 ± 0.05 ^d^	0.26 ± 0.02 ^e^	0.21 ± 0.01 ^f^
True density (g/mL)	2.85 ± 0.06 ^b^	2.94 ± 0.12 ^b^	2.95 ± 0.09 ^b^	1.04 ± 0.18 ^ef^	1.32 ± 0.12 ^de^	1.74 ± 0.25 ^cd^	1.95 ± 0.05 ^c^
Hardness (N)	67.45 ± 20.54 ^b^	65.64 ± 12.77 ^b^	66.01 ± 16.16 ^b^	44.14 ± 4.41 ^c^	40.33 ± 5.65 ^c^	24.52 ± 4.52 ^d^	24.55 ± 2.48 ^d^
First peak force (N)	51.69 ± 2.99 ^b^	52.81 ± 6.52 ^b^	50.47 ± 10.14 ^b^	34.09 ± 1.69 ^c^	14.07 ± 2.83 ^d^	10.51 ± 1.35 ^e^	9.98 ± 0.78 ^e^
WSI (%)	5.95 ± 0.47 ^g^	6.96 ± 0.25 ^f^	7.98 ± 0.12 ^de^	8.01 ± 0.37 ^cd^	8.26 ± 0.71 ^bd^	8.90 ± 0.47 ^bc^	9.01 ± 0.45 ^b^

^a^ L*, a*, b*, *C*, *H* and ΔE* represent the whiteness, redness, yellowness, chroma, hue angle, and total color difference, respectively. WSI is the water solubility index. ^b–g^ values bearing different superscript lowercase letters in the same row are significantly different (*p* < 0.05). − means not calculated. These tests were performed five times.

**Table 2:** The relative crystallinity, ratio of 1047/1020 (cm^−1^) and pasting properties of cassava starch under varying puffing times through sand-frying.

Parameters ^a^	Puffing Time (s)			
	0	6	12	18
RC (%)	7.06 ± 0.21 ^b^	3.49 ± 0.42 ^c^	0.38 ± 0.08 ^d^	0.31 ± 0.04 ^e^
1047/1020 (cm^−1^)	0.88 ± 0.01 ^b^	0.83 ± 0.00 ^c^	0.79 ± 0.00 ^d^	0.78 ± 0.00 ^d^
PV (cP)	5655 ± 85 ^b^	5134 ± 35 ^c^	3208 ± 211 ^d^	2639 ± 225 ^e^
TV (cP)	3226 ± 69 ^b^	3111 ± 53 ^b^	1962 ± 292 ^c^	1910 ± 35 ^c^
BD (cP)	2429 ± 47 ^b^	2023 ± 53 ^c^	1245 ± 275 ^d^	729 ± 191 ^d^
FV (cP)	4730 ± 49 ^b^	4611 ± 19 ^c^	3781 ± 178 ^d^	3256 ± 124 ^e^
SB (cP)	1504 ± 49 ^bd^	1499 ± 50 ^bc^	1818 ± 199 ^b^	1346 ± 108 ^cd^
PT_emp_ (°C)	50.12 ± 0.03 ^b^	50.23 ± 0.14 ^b^	50.27 ± 0.16 ^b^	50.57 ± 0.52 ^b^
PT (min)	4.16 ± 0.04 ^cd^	4.47 ± 0.07 ^bd^	4.53 ± 0.13 ^b^	4.51 ± 0.23 ^bc^

^a^ RC is the relative crystallinity. 1047/1020 (cm^−1^) means the ratio of 1047 to 1020 cm^−1^ in deconvo luted infrared spectra. PV, TV, BD, FV, and SB, respectively, represent the peak viscosity, through viscosity, breakdown (PV–TV), final viscosity, setback (FV–TV). PT_emp_ is pasting temperature and PT is peak time. ^b–e^ Values bearing different superscript lowercase letters in the same row are significantly different (*p* < 0.05). These tests were performed three times.

**Table 3:** The molecular weight of cassava starch and the branch chain length distribution of its amylopectin under varying puffing times by sand-frying.

Parameters ^a^	Puffing Time (s)			
	0	6	12	18
*M_w_* (kDa)	94,574.5	44,506.9	35,144.1	33,769.2
*M_n_* (kDa)	20,350.9	10,378.8	8829.1	7182.1
DP 6–12 (A)	32.3	34.5	35.2	35.9
DP 13–24 (B_1_)	49.7	50.4	52.3	53.3
DP 25–36 (B_2_)	11.1	10.3	9.6	8.2
DP ≥ 37 (B_3_)	6.9	4.8	2.9	2.6
*CL*	18.4	17.4	16.9	16.6

^a^ *M_w_* and *M_n_* are respectively known as weight- and number-average molecular weight. DP and *CL* are the degree of polymerization and average chain length, respectively.

## Data Availability

The data presented in this study are available on request from the corresponding author.

## Supplementary Materials

The following are available online at https://www.mdpi.com/article/10.3390/biom11121872/s1, Figure S1: The appearance changes of dried cassava starch gel under various puffing times by sand-frying. Figure S2: The glass transition temperature of the dried starch disc. Figure S3: The pore size distribution of dried cassava starch gel at puffing time of 9s (A), 12s (B), 15s (C), and 18s (D) by sand-frying. Figure S4: Fourier transforms infrared spectra of cassava starch at varying puffing times by sand-frying. Table S1: Proximate composition of cassava starch (g/100 g). Video S1: The preparation of puffed starch gel.
