# Supplementary material for: Effect of Sand-Frying-Triggered Puffing on the Multi-Scale Structure and Physicochemical Properties of Cassava Starch in Dry Gel
Source: Biomolecules. 2021 Dec 14;11(12):1872. doi: 10.3390/biom11121872 (PMC8699278; doi:10.3390/biom11121872)
Supplement: Supplementary file 1 [file biomolecules-11-01872-s001.zip › biomolecules-1480956-final-suppl/Supplementary Figures and Tables.pdf]

Supplementary Materials

Effect of Sand-frying-triggered Puffing on the Multi-scale Structure and Phyicochemical Properties of Cassava Starch in Dry Gel

Yonglin He [1], Fayin Ye [1], Sheng Li [1], Damao Wang [1], Jia Chen [1], Guohua Zhao [1,2*]

[1] College of Food Science, Southwest University, Chongqing 400715, People's Republic of China

[2] Chongqing Engineering Research Center for Sweet Potato, Chongqing 400715, People's Republic of China

*Corresponding author: Guohua Zhao

E-mail address: zhaogh@swu.edu.cn

Address: College of Food Science, Southwest University, 2 Tiansheng Road, Chongqing, 400715, People's Republic of China

Tel: 86-23 68252118

Supplementary Figures

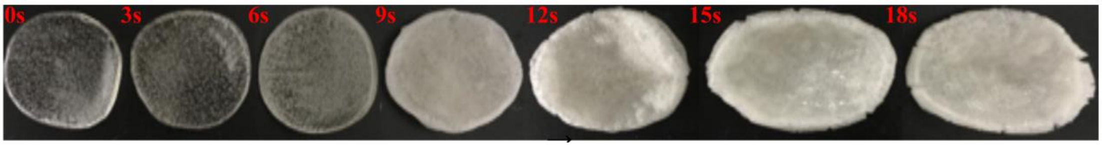

Figure S1. The appearance changes of dried cassava starch gel under various puffing time by sand-frying.

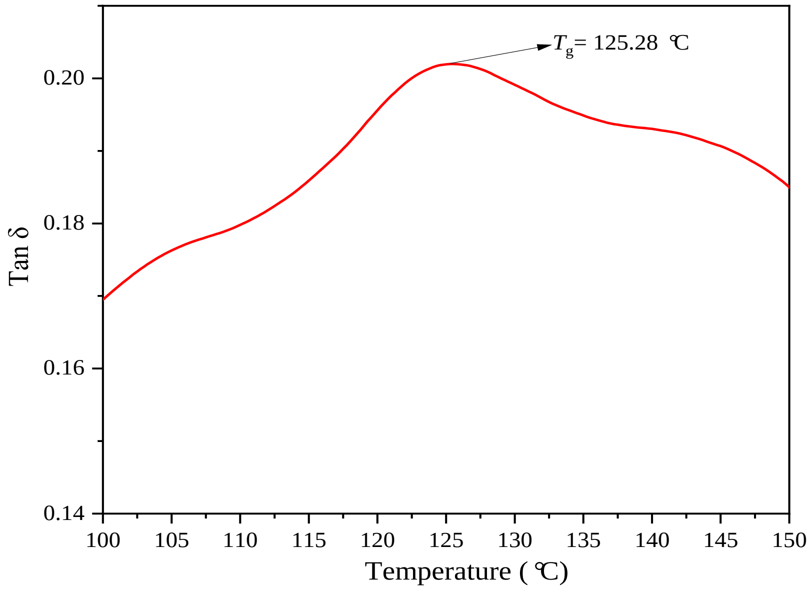

Figure S2. The glass transition temperature of dried starch disc.

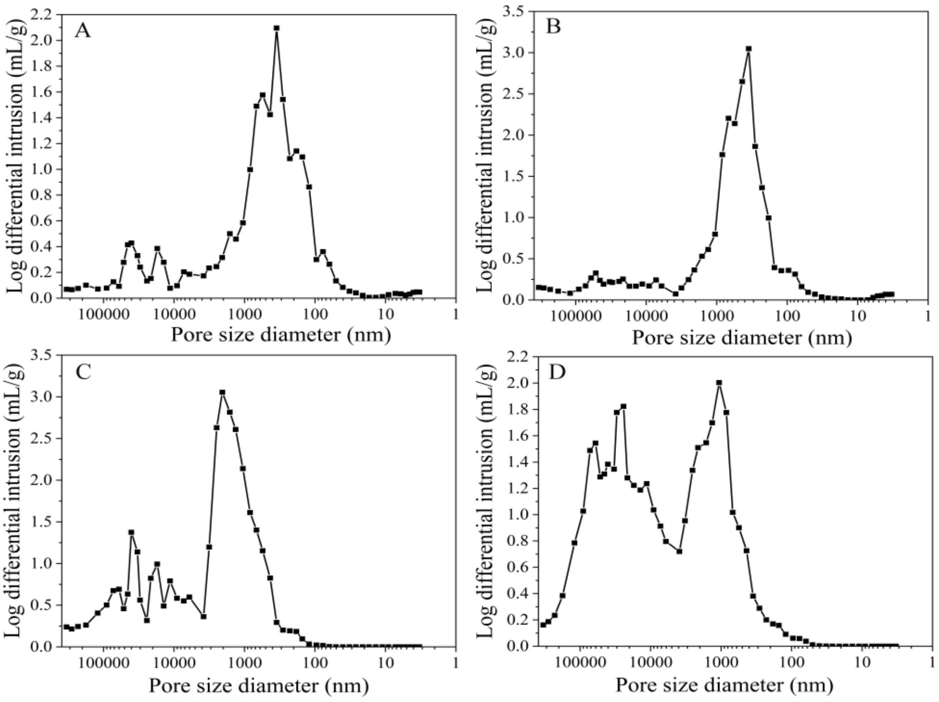

Figure S3. The pore size distribution of dried cassava starch gel at puffing time of 9s (A), 12s (B), 15s (C) and 18s (D) by sand-frying, respectively.

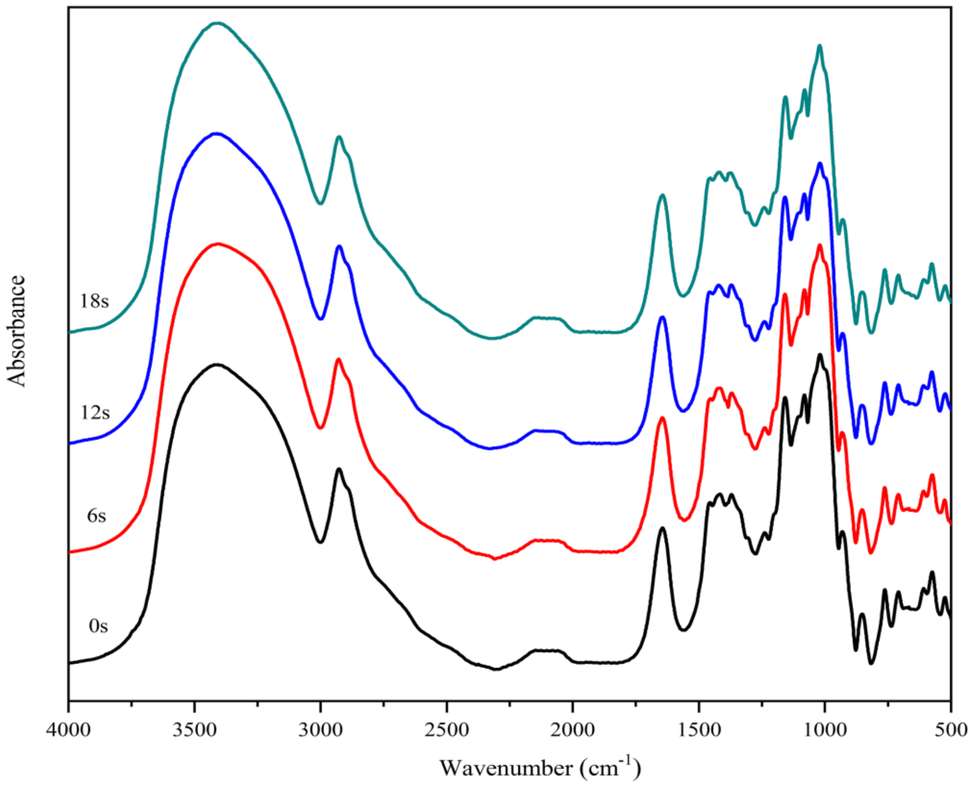

Figure S4. Fourier transform infrared spectra of cassava starch at varying puffing time by sand-frying.

Supplementary Tables

Table S1. Proximate composition of cassava starch (g/100g).

Starch Origin	Starch	Amylose	Protein	Lipid	Moisture	Ash
Cassava	85.21 ± 1.52	19.42 ± 1.13	0.29 ± 0.01	0.19 ± 0.02	13.91 ± 0.21	0.28 ± 0.01
